# What is simulation-based medical education (SBME) debriefing in prehospital medicine? A qualitative, ethnographic study exploring SBME debriefing in prehospital medical education

**DOI:** 10.1186/s12909-023-04592-8

**Published:** 2023-09-03

**Authors:** Maria Ahmad, Michael Page, Danë Goodsman

**Affiliations:** Barts and the London School of Medicine and Dentistry, Institute of Health Sciences Education, Queen Mary University of London, Turner Street, Whitechapel, London, E1 2AD UK

**Keywords:** Prehospital care, Education, Teaching, Simulation, Debriefing

## Abstract

**Introduction:**

Simulation-based medical education (SBME) debriefing – a construct distinct from clinical debriefing – is used following simulated scenarios and is central to learning and development in fields ranging from aviation to emergency medicine. However, little research into SBME debriefing in prehospital medicine exists. This qualitative study explored the facilitation and effects of prehospital SBME debriefing, and identified obstacles to debriefing, using the London’s Air Ambulance Pre-Hospital Care Course (PHCC) as a model.

**Method:**

Ethnographic observations of moulages and debriefs were conducted over two consecutive days of the PHCC in October 2019. Detailed contemporaneous field notes were made and analysed thematically. Subsequently, seven one-to-one, semi-structured interviews were conducted with four PHCC debrief facilitators and three course participants to explore their experiences of prehospital SBME debriefing. Interview data were transcribed and analysed thematically.

**Results:**

Four overarching themes were identified: approach to facilitation of debriefs, effects of debriefing, facilitator development, and obstacles to debriefing.

The unpredictable debriefing environment was seen as both hindering and, paradoxically, benefitting SBME debriefing. Despite using varied debriefing structures, facilitators emphasised similar key debriefing components including exploring participants’ reasoning and sharing experiences to improve learning and prevent future errors.

Debriefing was associated with three effects: releasing emotion; learning and improving, particularly compound learning as participants progressed through sequential scenarios; and the application of learning to clinical practice. Facilitator training and feedback were central to facilitator learning and development.

Several obstacles to debriefing were identified, including mismatch of participant and facilitator agendas, pressure and time.

**Conclusions:**

SBME debriefing in prehospital medicine is complex, requiring an understanding of participant agendas and facilitator experience to maximise participant learning. Aspects unique to prehospital SBME debriefing were identified, notably, the unpredictable debriefing environment, and the paradoxical benefit of educational obstacles for learning. Aspects of SBME debriefing not extensively detailed in the literature were also highlighted, such as compound participant learning, facilitator candour, and facilitator learning, which require further exploration.

## Background

Debriefing, as defined by Allen et al.—*“a type of work meeting in which teams discuss, interpret, and learn from recent events during which they collaborated*” [1]—originated in the military and has since been utilised in aviation, business, and healthcare, with the defining features across all contexts being discussion, reflection and learning [1]. It is particularly valuable in the emergency and prehospital setting due to the ability to discuss and prepare for common, time-critical scenarios [2], and has been shown to improve participants’ technical and non-technical skills [3–5].

Simulation-based medical education (SBME) debriefing is a specific subset of debriefing practices (see Table 1 for examples) that is distinct from non-SBME debriefing approaches, such as operational or therapeutic debriefing, as it foregrounds participant reflection and learning [5]. Although a systematic review by Issenberg et al. identified debriefing as the most important aspect of simulation-based medical education, [6] there has been little exploration of SBME debriefing in prehospital medicine [5], with the literature often focusing on the simulated scenarios rather than the debrief.

**Table 1:** Key debriefing approaches and structures

Model and description	Example
**Pendleton **[7] The facilitator asks the learner what they did well, before adding more positive points. The same is done for potential improvements	*“What went well?”* *“What could you do better or differently next time?”*
**The feedback sandwich **[8] Positive feedback is given initially, followed by constructive feedback, finally ending with more positive feedback. Therefore, the more negative feedback is “sandwiched” in the middle of positive feedback	*“Your rapid A-E assessment was excellent and allowed you to quickly identify the wheezing. However, you needed to treat the wheezing initially with salbutamol, rather than starting with magnesium. This was a challenging scenario but you did well.”*
**Advocacy-inquiry **[9] Observation or statement (advocacy) followed by a question (inquiry). A form of ‘debriefing with good judgement’	*“I noticed that when the patient began wheezing, you gave magnesium. Usually we would start with salbutamol [advocacy]. Why did you decide to give magnesium first? [inquiry]”*
**Promoting Excellence and Reflective Learning in Simulation (PEARLS) **[10] A debriefing model that promotes the intentional use of multiple debriefing strategies within one model or debriefing event, which is tailored to participant needs (blended debriefing)	*Reactions phase – allow participants to release emotions* *Description phase – clarify the scenario* *Analysis phase – select an appropriate debriefing approach considering the time available, the content area to be explored, and whether the rationale for the knowledge gap is clear* *Application and summary*

Furthermore, the prehospital SBME debriefing setting is unlike that of other emergency or critical care specialities, as it comprises a different physical environment, way of working and culture to that with which most clinicians are accustomed, which is important given the vital role of context within medical education [11]. It is possible, therefore that SBME debriefing in pre-hospital medicine gives rise to additional, corollary benefits alongside reflection and learning, and constitutes an educational practice that is fundamentally different to SBME debriefing in other medical education settings, including other emergency medicine settings.

Consequently, we set out to answer the research question, ‘What is SBME debriefing in prehospital medical education?’ and the related question, ‘What are its effects?’ We did so through four salient sub-questions, which were developed after initial observations of the course: How did faculty effectively facilitate SBME debriefs? What were the effects of their approaches? How did facilitators develop through debriefing? What were the obstacles to SBME debriefing? As few studies in the literature explore these research questions [5], this study contributes to an understanding of current prehospital SBME debriefing practices and how to improve debrief facilitation, thus enhancing participant learning and prehospital clinical practice.

## Methodology

### Background to moulages on the PHCC

London’s Air Ambulance (LAA) is a prehospital service that employs Helicopter Emergency Medical Service (HEMS) doctors and paramedics. As part of their induction for working with LAA, clinicians must complete an intensive seven-day course known as the Pre-Hospital Care Course (PHCC). Additionally, the course attracts delegates from HEMS services across the UK and internationally. Course participants have years of clinical experience, with most participants being senior paramedics, or emergency medicine or anaesthetic registrars or consultants, and have therefore participated in, and sometimes led, numerous debriefs. The course comprises lectures from prehospital experts alongside moulages – simulated scenarios utilising makeup and special effects to simulate a range of illnesses or injuries on mannequins and simulated patients – and SBME debriefs by facilitators, who are current or previous HEMS doctors or paramedics [12, 13]. Debriefing is a key part of the educational process of the PHCC.

Several days of moulages, immediately followed by their respective debriefs, take place on the PHCC. The moulages take place both indoors and outdoors and cover a wide range of traumatic presentations requiring specific HEMS skills. Moulages are based on previous cases attended by LAA, to provide realistic situations that closely mimic real prehospital environments, patient presentations and challenges. Typically, four moulages and the associated debriefs take place per session, with participant groups rotating around the different moulages while facilitators are allocated to run a specific moulage for the duration of the session. Moulages are typically 10–20 min in duration, with debriefs lasting a similar length of time.

### Method

A mixed methods qualitative ethnographic approach, through course observation and interviews, was chosen to gain an in-depth insight into the lived experience of PHCC participants and facilitators. This approach was chosen as ethnography can provide insights into culturally embedded practices that may not be evident through interviews alone [14]. This is because people ‘do not or cannot always describe what they actually do and think during an interview’ (p. 1). However, observations can offer insights that illuminate interview participants’ narratives, offering a more complete understanding of the phenomenon at the heart of the research. Our prior knowledge of the PHCC suggested that, particularly for course participants, the pressure of the ‘hot’ debriefing experience may lead to differences between what was actually happening during the debriefs and their recollection of the debriefs some time later. Thus, observations added a degree of methodological triangulation to the process of data collection and interpretation, and was used to increase the richness of the data [15]. We also felt that observations were important for us as researchers to understand the ‘shorthand’ – the various turns of phrase or references – used by interview participants when discussing the programme^1^.

The PHCC moulages and debriefs were observed over two consecutive afternoons in October 2019 by MA. MA was not personally known to participants during the observational phase, but they were aware of MA’s research study and interests, and consented to be observed. Observational data were generated through detailed contemporaneous field notes made while following one group of PHCC participants through the moulages and debriefs, noting the discussions, body language and reactions of facilitators and participants. Despite overt observation, this was unlikely to have impacted the behaviour of those being watched as the PHCC regularly has external observers, peers and facilitators observing, and prehospital clinicians are used to being in the public eye due to the nature of prehospital medicine.

Prior to contacting potential interviewees, ethical approval was obtained from Queen Mary University of London. Purposive sampling was used to identify nine clinicians who had previously been observed. All were contacted by email, with two not responding. Therefore, seven clinicians, comprising four PHCC facilitators and three participants, with a range of clinical and educational experience were chosen (Table 2).

**Table 2:** Overview of the clinical and SBME debriefing experiences of interviewees

Interviewee	Amount of total clinical experience (years)	Type of predominant clinical experience(s)	Prehospital clinical experience (years)	SBME debrief facilitation experience (years)
Facilitator 1	13	Doctor	1	6
Facilitator 2	32	Paramedic	22	15
Facilitator 3	32	Paramedic	25	20
Facilitator 4	21	Doctor	14	11
Participant 1	12	Doctor	0.5	0.5
Participant 2	6	Doctor	1	3
Participant 3	11	Doctor	4	1.5

One-to-one semi-structured interviews were chosen to allow exploration of facilitators’ and participants’ perceptions of SBME debriefing on the PHCC. Prior to commencing interviews, a verbal explanation and a participant information sheet were given, and participant consent forms were signed. Interviews were carried out between 30^th^ December 2019 and 29^th^ February 2020 by MA on university campus or in hospital offices, and ranged between 25 and 93 min. No non-participants were present. An interview guide, informed by the observational data previously gathered, was created to aid the interview but was not prescriptive, and brief field notes were made during and after each interview. Following manual transcription of the interviews and a playback of the audio to check transcripts for accuracy, audio recordings were deleted. All data were anonymised and interview transcripts were kept securely. Participants did not request transcripts or research findings, and no repeat interviews were carried out. Data saturation, a concept drawn from grounded theory research, was not used here but was it clear that after seven interviews no new concepts were emerging.

**Fig. 1:**
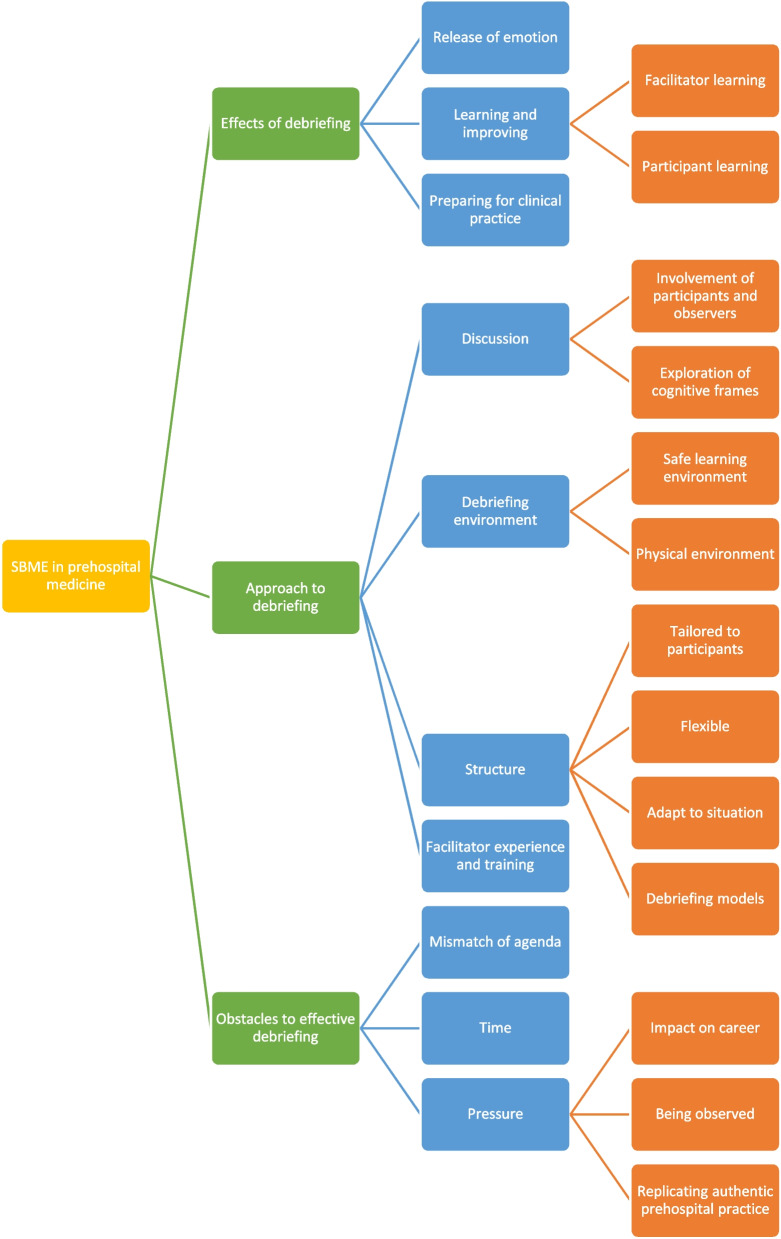
Initial coding framework developed by MA. Several rounds of discussion were used to identify main themes

Significant data were generated, comprising 9 h of course observation and 5.5 h of interviews. Thematic analysis of the combined data set, following the process described by Braun and Clarke [16], was used:Data familiarisation was undertaken by MA and MP, reading and re-reading the transcripts multiple times. MA and MP independently generated initial codes, which were discussed and agreed. MA identified themes and constructed an initial coding framework (Fig. 1), and the themes and framework were reviewed by MA and MP. Several rounds of discussion were used to refine the themes and determine those with greatest significance, and MA applied the coding framework to the whole of the data set.

## Results and discussion

Observational and interview data were organised into four overarching themes: i), the approach to facilitation of debriefs, ii), the effects of debriefing, iii), facilitator development, and iv), obstacles to effective SBME debriefing in prehospital medicine. To strengthen the links between the themes identified, the data collected and its relation to the wider literature, the results and discussion have been presented here together.

### Approach to facilitation of SBME debriefs

#### The debriefing environment

Interview and observational data stressed the importance of facilitators optimising the physical debriefing environment.

*“Are you going to get the best out of somebody who’s sitting outside when it’s cold? No, you need to get them in the warm with a cup of tea, you need to make it conducive to people wanting to contribute to it.”*

Facilitator 2

This highlights that facilitators making the physical debriefing environment comfortable and welcoming may help to create an open learning environment and increase participant engagement.

Interviewees felt that the environment should be *“as private as possible”* (Participant 3) to allow participants to feel at ease and discuss their thoughts in a confidential learning environment. Field notes highlighted that facilitators moved participants away from the scene to debrief, creating a symbolic distance from the stress of the educational scenario, mirroring the typical approach in prehospital clinical practice.

Interviewees also suggested that relationships between team members contributed to the supportive learning environment through a sense of belonging.

*“There's a nice team mentality…you come together as a group and support each other.”*

Participant 3

**Fig. 2:**
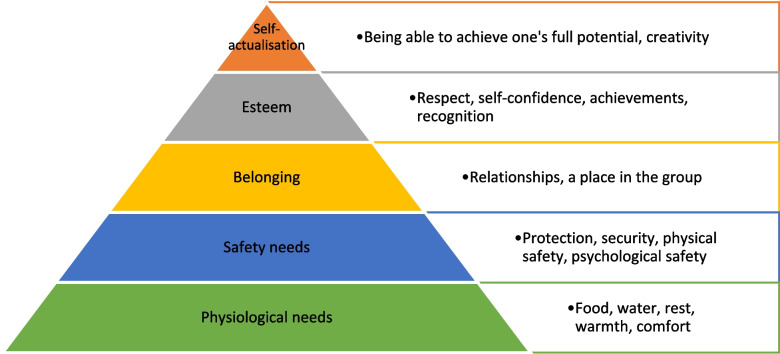
Diagrammatic representation of Maslow's hierarchy of needs in relation to the debriefing environment. Adapted from Maslow [17]

Therefore, viewing the debriefing environment through the lens of humanistic psychology [17] (Fig. 2) it appears that participants’ physiological needs in terms of the physical environment, psychological safety through the safe learning environment, belonging, and esteem are important considerations [17], and that appropriate curation of the debriefing environment by facilitators lays the foundation for effective debriefing and participant learning.

However, there is added complexity in prehospital SBME debriefing as noise and distractions, which are largely eliminated in hospital SBME debriefing, are key to enhancing scenario and debrief fidelity. Ethnographic observation revealed that moulages and debriefs were often conducted outside, near an active helipad, making it challenging for participants to listen and contribute, or interrupting the flow of debriefs. The ideal physical debriefing environment is therefore not always possible in prehospital medical education. However, there may be a paradoxical benefit, in that this unpredictable and uncontrolled environment – one of the defining characteristics of prehospital medicine and thus important for scenario fidelity – may authentically reflect the challenges of debriefing in prehospital clinical practice.

#### Structure and adaptability

Although facilitators used a range of debriefing structures, from Pendleton [7] to advocacy-inquiry [9], many emphasised similar core components. These included: an initial reaction phase, summarising scenario events, discussion of participant actions and reasoning in the scenario, summarising key learning points, and application to clinical practice. These align with common features of debriefing structures across the literature [10, 18, 19].

Although participants perceiv a greater benefit to their learning and clinical skills following structured debriefing sessions [20], interviewees felt that a rigidly defined debriefing structure *“protocolised”* debriefing and made it *“[less] organic”*(Participant 3). This suggests that a reflexive or dynamic approach such as PEARLS [10], may be most suited to debriefing in this context.

#### Exploration of cognitive frames

A cognitive frame refers to how participants perceive and make sense of a scenario, which thus influences their actions. Therefore, misunderstandings and mistakes often result from how each participant frames the situation, which can be uncovered through discussion in the debrief [9, 21].

All facilitators highlighted the importance of understanding participants’ cognitive frames. Exploration of these during the debrief, often through advocacy-inquiry [9], helped participants reflect on their performance, identifying and correcting errors to improve their technical skills. This was particularly useful in the prehospital SBME debriefing setting as the experienced participants were able to closely reflect on their own performance and that of their peers, and identify important learning points.

When frames were not explored, participants felt frustrated that their learning had been limited.

*“There wasn’t much opportunity for us to explore how we were feeling at that point or what had led us to those decisions, it was quite a closed discussion and that felt really unfulfilling because we weren’t able to express why we’d got there.”*

Participant 2

#### Learning from shared experience

Facilitators often shared their personal experiences with participants, which Facilitator 3 felt made facilitators *“more approachable human[s].”* In particular, participants felt *“supported to share”* when facilitators *“[shared] where they made a mistake”*(Participant 3). This candour allowed facilitators to build trust with participants, increasing participant engagement and reducing their anxiety about making mistakes. This technique was highly effective, but has had little exploration in the literature elsewhere, perhaps because it is key to the prehospital just culture in LAA which embodies sharing collective learning through team and organisational reflection [22], allowing participants and facilitators to feel they can share previous experiences and mistakes openly.

Participants valued practical recommendations relating to clinical practice from facilitators, given their experience in world-renowned prehospital services.

*“That real-world nous and advice that comes from hard-won experience is the most valuable kind.”*

Participant 3

Participants felt the advice imparted through debriefs, and the experiences shared by scenario participants and observers had *“saved so much clinically relevant time on scene”* (Participant 3), improving clinical performance and patient care.

### Effects of debriefing

#### Releasing emotion

Facilitators emphasised the beneficial effect of debriefing in allowing participants to express their feelings and *“get stuff off their chest”* (Facilitator 1) following challenging scenarios. Without this emotional release, participants left feeling *“frustrated”* and *“worried”*, which *“put a cap on learning from the debrief”* (Participant 2). This highlights that allowing participants time and space to initially express their emotions through the ‘reaction phase’ in debriefing models [10, 18, 19, 23] can reveal their worries, which can then be addressed, guiding the remainder of the debrief and allowing them to focus on learning [18, 23]. This is particularly relevant in the prehospital debriefing setting where the scenarios were described as *“a lot more volatile, a lot more challenging and a lot more emotive…much more intense than the traditional life support and in-hospital courses.”*(Participant 2). We note that this is in contrast to some debriefing approaches that explicitly aim to side-line or ‘park’ emotions [24]. However, the important interaction between emotions and cognition [25] suggests that it may be a false economy to try to place emotion on hold in order to prioritise a more dispassionate analysis.

#### Learning and improving

Participants felt that the debrief was *“where the real value is”*(Participant 3), often being more useful for learning, improvement and application to clinical practice than the scenario itself, as supported by multiple studies and educational theories [3, 4, 26, 27]. The debrief was considered *“part of the [prehospital] culture, it’s just normal for us to do it…[although] it might seem really alien if you’re coming the first time”* (Participant 3). This illustrates that debriefing is embedded within the culture of prehospital clinical practice and medical education such that it has become an expected part of prehospital medicine, much more so than for other medical specialities.

Observational data illustrated that participants were constantly drawing from and building on their prehospital experience, skills and knowledge when participating in scenarios and debriefs, resulting in continuous improvement as they progressed through scenarios. For example, one participant commented, “*If you asked me a week ago* [about the diagnosis] *I would have said haemorrhagic shock, but now I’ve learnt about bleeding mimics so was looking out for them too.*” (Observation Day 2, Debrief following Moulage 2). We termed this cumulative learning process *compound learning*. This is supported by constructivist learning theory, which states that learners develop understanding by drawing on existing knowledge and combining it with the new knowledge and ideas they encounter [28]. Therefore, understanding the previous scenarios and debriefs participants have had, and their likely compound learning, helps facilitators guide their debrief.

#### Preparing for clinical practice

PHCC participants described long-term transfer to clinical practice, and attributed this to the fidelity of the scenarios and the quality of the debriefs, which helped them discuss and develop *“strategies for managing the scene”* (Facilitator 3).

Additionally, HEMS clinicians are expected to debrief the team following real clinical situations.

*“Having done the PHCC I know what I’m looking for and what aspects to discuss.”*

Participant 1

Therefore, exposing PHCC participants to varied debriefing styles allowed them to understand how to debrief a team through role-modelling [29].

### Facilitator development

#### Facilitator training

Currently, LAA facilitator training appears to follow an informal experiential learning approach, with facilitators using their lived experience of participating in hundreds of moulages and debriefs to facilitate them. Tariq et al. believe this informal learning is beneficial due to the extensive knowledge LAA clinicians already have. However, Facilitator 2 – a senior clinician in charge of education governance – explained:

*“Although good people will just pick it up by watching others I think there’s a gamble with that…Teaching is a powerful tool, people will listen to what you say. If you say the wrong thing…you can damage people…So I’ve kind of talked myself into the fact you need a training course.”*

Facilitator 2

Time was the main barrier to building formal debriefing training into LAA. To manage this, Paige et al. suggest incorporating debriefing training into the continuing professional development framework [30].

Facilitator 4 highlighted the challenge of providing feedback to highly clinically experienced facilitators. Several frameworks have been developed to provide objective feedback to facilitators [31–33], and prehospital training courses may benefit from making greater use of these.

#### Facilitator learning

Facilitators learnt and improved from facilitating scenarios and debriefs, through self-reflection and discussion with participants, with Facilitator 2 expressing that he was learning “*all the time”,* such as after the following scenario of a patient with haemorrhagic shock.

*Facilitator: It felt like you just did the central line because you had nothing to do.*

*Participant: But the two cannulas were pink, I couldn’t put blood through that.*

*Facilitator: Actually, that’s a good point. We’ll change that for future scenarios.*

Observation Day 1, Debrief following Moulage 1.

Through discussion of participants’ cognitive frames, facilitators learnt that they needed to change the cannula size to improve scenario fidelity, which they adopted for future scenarios. This phenomenon of facilitator learning from exploration of cognitive frames has had limited exploration in the literature.

To enhance facilitator learning in the prehospital debriefing setting, course faculty teams should consider implementing formal individualised feedback from participants, to promote discussion and reflection by facilitators of their debriefing.

### Obstacles to effective SBME debriefing

#### Mismatch of agendas

Facilitators and participants sometimes held conflicting opinions regarding what should be discussed during the debrief. Participant 2 speaks about a debrief which he left feeling frustrated due to this agenda mismatch.

*“It was clear the facilitator for that scenario wanted to talk about the technical points, whereas my ‘agenda’ for that debrief was less on the technical and more on the non-technical points.”*

Participant 2

This suggests that understanding the different agendas of participants and facilitators is essential for the success of the debrief. By encouraging participants and facilitators to express what they wish to discuss at the beginning of the debrief, both parties can agree the balance between their agendas, setting the focus of the debrief.

#### Pressure

All interviewees highlighted the pressure of performing while being observed and assessed by esteemed facilitators and peers on the PHCC.

*“There’s a lot of expectation…you feel to some degree judged by people…Right at the start of your career, you're exposing yourself to looking foolish by doing something wrong.”*

Participant 3

Despite being experienced clinicians who perform in front of relatives, peers and seniors every day, PHCC participants worry about the impact on their career if they make mistakes, due to the perceived expectations and judgements of their facilitators and peers, who will be their future colleagues. This may negatively impact their self-confidence, destroy the safe learning environment and reduce the richness of learning gained from mistakes [34, 35]. This feeling of being *“under the microscope”* (Participant 1) aligns with research by Savoldelli et al. who found that simulation and debriefing can give rise to a vulnerable psychological state in participants who fear the judgement of the facilitator and their peers, thus presenting a barrier to learning [36].

However, the pressure experienced by participants may be beneficial in the long-term through simulating *“real world stress”*(Participant 3), such as from a rapidly deteriorating patient, while the pressure of high expectations may translate to increased participant motivation and improved performance [37].

#### Time

Time was the biggest obstacle to effective debriefing, and was mentioned on average ten times per interview. Our research suggested that time may have impacted the ability of facilitators to address participant agendas, tailor the debrief to the participant group and train facilitators.

*“If the scenario has run on, you know the debrief is going to have to be rushed…you’re going to have to do more of a rapid-fire, taking control of the debrief.”*

Participant 2

Time constraints sometimes led to participant frustration from reduced involvement and mismatched agendas. One solution could be setting the time allocated to debriefing to double that for the scenario [23]. However, some interviewees felt time constraints *“focused”* (Facilitator 2) the learning from the debrief, helping participants better remember the crucial learning points for the future and preventing information saturation.

### Research strengths and limitations

Limitations of the work included the fact that all interview participants were male, which was not intended when initially recruiting participants. This may represent the wider gender imbalance within prehospital medicine; only 8 of 31 LAA clinicians were female at the time of research [38]. As paramedics were unavailable, more doctors were chosen, however, twice as many HEMS doctors as paramedics were working at LAA at the time of research [38].

As only a small sample was interviewed from one PHCC, their views may not be generalisable to all PHCC facilitators and participants. Additionally, observation for the entire duration of the PHCC would allow further investigation of the facilitator-participant dynamic, facilitator learning, and participant compound learning. Although many international courses have been built on the PHCC model, those in other prehospital services are likely to have different methods of and obstacles to debrief facilitation, which are worth exploration.

Nonetheless, we believe that research is credible, as it exhibits several strengths. These include the mixed methods approach, which combined naturalistic observations and interview data, and the ethnographic methodology which took account of the exigencies of an authentic, highly dynamic educational setting. Given the practical nature of the findings, we believe that they can be used by practitioners to enhance their own prehospital medicine simulation programmes.

## Conclusion

SBME debriefing in prehospital medicine was revealed to be complex, requiring a safe learning environment, an understanding of participant agendas, and facilitator experience to maximise participant learning through the exploration of participant cognitive frames and flexible, learner-led approaches, within time constraints. Aspects unique to prehospital SBME debriefing were identified, notably, the unpredictable debriefing environment, and the paradoxical benefit of educational obstacles for learning. Aspects of SBME debriefing not extensively detailed in the literature were also highlighted, such as compound participant learning, facilitator candour, and facilitator learning, which require further exploration.

Obstacles to effective SBME debriefing, including mismatch of agendas, performance pressure and the impact of unaddressed emotional reactions, were highlighted. Interestingly, some of these obstacles, most notably performance pressure and time constraints, could paradoxically enhance authenticity and thereby potentially improve participant learning when used appropriately within the SBME environment. For some, these mismatches were seen as an authentic reflection of the environment and an opportunity for broader learning. Therefore, we recommend that prehospital courses consider professional development for facilitators to highlight the importance of understanding participants’ perspectives and agendas and foregrounding the emotional aspects of simulation participation within the debrief. Formal facilitator feedback as part of the course would also help to enhance the delivery of SBME debriefs and allow faculty teams to highlight and address obstacles.

We suggest that future research should further explore our unique findings, in particular, the compound participant learning, facilitator candour, facilitator learning and the paradoxical benefit of educational obstacles, to evaluate their applicability to prehospital SBME debriefing in courses beyond LAA. We also feel that the role of the observer in these scenarios merits further exploration.

## Data Availability

The datasets generated and analysed during the current study are not publicly available as we do not have participant consent for this but are available from the corresponding author on reasonable request.

## Abbreviations

PHCC: Pre-Hospital Care Course
LAA: London’s Air Ambulance
HEMS: Helicopter Emergency Medical Service
PEARLS: Promoting Excellence and Reflective Learning in Simulation
PHEM: Pre-Hospital Emergency Medicine
EM: Emergency Medicine
ICU: Intensive Care Unit

## Footnotes

1 An example of this shorthand is participants’ use of the phrase ‘one under,’ to refer to a patient who is trapped under a train. We found that there was a whole prehospital professional lexicon with which it was important to become familiar in order to fully understand the context, and participants’ reflections on it.
